# A novel P indicator to evaluate bread wheat (*Triticum aestivum*) genotypes to identify tolerance to phosphorus deficiency based on two distinct root phenotyping platforms

**DOI:** 10.1093/aob/mcaf091

**Published:** 2025-06-10

**Authors:** Fabiano Sillo, Christophe Salon, Mickael Lamboeuf, Vincenzo Montesano, Stephan Summerer, Angelo Petrozza, Adriano Conte, Francesco Bergese, Francesca Degan, Raffaella Balestrini, Christian Jeudy

**Affiliations:** National Research Council of Italy, Institute for Sustainable Plant Protection (CNR-IPSP), Strada delle Cacce 73, 10135 Torino, Italy; INRAE, UMR 1347 Agroécologie, 17 Rue Sully, BP 86510, 21065 Dijon Cedex, France; Plant Phenotyping Platform for Plant and Micro Organisms Interactions (4PMI), 17 Rue Sully, BP 86510, 21065 Dijon Cedex, France; INRAE, UMR 1347 Agroécologie, 17 Rue Sully, BP 86510, 21065 Dijon Cedex, France; Plant Phenotyping Platform for Plant and Micro Organisms Interactions (4PMI), 17 Rue Sully, BP 86510, 21065 Dijon Cedex, France; National Research Council of Italy, Institute for Sustainable Plant Protection (CNR-IPSP), Strada delle Cacce 73, 10135 Torino, Italy; ALSIA Centro Ricerche Metapontum Agrobios, S.S. Jonica 106, km 448.2, 75010 Metaponto di Bernalda, Italy; ALSIA Centro Ricerche Metapontum Agrobios, S.S. Jonica 106, km 448.2, 75010 Metaponto di Bernalda, Italy; National Research Council of Italy, Institute for Sustainable Plant Protection (CNR-IPSP), Strada delle Cacce 73, 10135 Torino, Italy; National Research Council of Italy, Institute for Sustainable Plant Protection (CNR-IPSP), Strada delle Cacce 73, 10135 Torino, Italy; ARVALIS, Station Expérimentale de Boigneville, 91720 Boigneville, France; National Research Council of Italy, Institute of Biosciences and Bioresources (CNR-IBBR), via Giovanni Amendola 165/A, 70126 Bari, Italy; INRAE, UMR 1347 Agroécologie, 17 Rue Sully, BP 86510, 21065 Dijon Cedex, France

**Keywords:** Cereals, wheat, *Triticum aestivum* L, phosphorous, phenotyping, root

## Abstract

**Background and Aims:**

Phosphorus (P) is a crucial macronutrient for plant growth that, despite its abundance in soils, is often a limiting factor in agricultural productivity, particularly for cereals such as wheat. In this study, the response of different wheat genotypes to two different levels of P was evaluated in a large trial encompassing 26 genotypes using two distinct root phenotyping platforms, ALSIA and 4PMI.

**Methods:**

Rhizotubes allowed non-invasive root phenotyping, revealing significant genotypic effects on biomass production and root system traits. Phosphorus acquisition and use efficiency of the wheat genotypes were estimated by using five different metrics.

**Key Results:**

A synthetic indicator for agronomic relevance integrating the efficiency metrics was established. Under optimal conditions, after 96 d, P acquisition efficiency (PAE) was inversely correlated with P utilization efficiency (PUE), suggesting an acquisition–use trade-off. Conversely, under low P conditions, both after 27 and 96 days PAE and PUE showed moderate positive correlations, indicating adaptive coordination to improve P utilization under scarcity.

**Conclusions:**

Overall, our findings highlighted the importance of root–target strategies in P efficiency in wheat, providing insights for breeders to enhance P deficiency tolerance in wheat.

## INTRODUCTION

Phosphorus (P) is one of the most challenging nutrients to manage in agricultural systems (Roberts and Johnston, 2015), fundamental to plant growth and development and key to a wide range of biological processes (Vance *et al*., 2003; Roberts and Johnston, 2015). It plays an indispensable role in energy transfer, structural integrity, signal transduction and a multitude of other vital cellular processes (Schachtman *et al*., 1998). Although total P in soil is abundant, plants can only take it up in the form of phosphate, which becomes rapidly unavailable because it is rapidly adsorbed and precipitated by binding with soil minerals, e.g. clay minerals, aluminium, iron and manganese oxides in acid soils and by calcium cations in alkaline soils (Shen *et al*., 2018). For this reason, fertilizers are commonly used to provide P to crops and cereals, which are among the world's most significant food sources and are highly dependent on adequate P supply. Cereal crops such as wheat, maize and rice are highly sensitive to P deficiency, which significantly reduces grain yield and quality (Dhillon *et al*., 2017). Among these crops, bread wheat (*Triticum aestivum* L.) is crucial for global food security, as it is known for its adaptability and dominance in cultivation worldwide and primarily used for making leavened and flatbreads, along with other baked products (Mastrangelo and Cattivelli, 2021). However, wheat relies heavily on P fertilizers due to its limited tolerance of stress due to low P, which adversely affects tiller production and head development (Dharmateja *et al*., 2021; Soumya *et al*., 2021). Thus, P fertilization presents a dual challenge, impacting both intensive agriculture in developed nations and extensive systems in developing regions. Phosphorus fertilizers, derived from rock phosphate and produced in limited areas, currently face the rising global demand driven by population growth and need for high food quality (Vance *et al*., 2003; Campos *et al*., 2018). Additionally, when fertilizers are applied, usually only 10–30 % of the P is taken up during the first year while a significant amount is accumulated in the soil as residual P not readily available for plants (Syers *et al*., 2008). In this context, it is crucial to identify and produce P-efficient germplasm, and promote agricultural practices that improve soil P accessibility for a sustainable approach to P in agriculture (Heuer *et al*., 2017).

Cultivating crops with improved P acquisition efficiency (PAE) and increased P use efficiency (PUE) is deemed to be a pivotal approach for boosting agricultural output (Vance *et al*., 2003; Lambers *et al*., 2006; Wang *et al*., 2010). It is known so far that variability in the ability of a plant to acquire soil P is determined by a combination of genetic and environmental factors (Nielsen and Schjørring, 1983). In nutrient limitation conditions, the reduction of the biomass is one of the major impacts, and particularly in low P conditions the most generalized symptoms among plant families are an overall reduction of P concentration in tissues and an increased root/shoot ratio (Chien *et al*., 2018; Wang *et al*., 2023). Plants exhibit various adaptation mechanisms to counteract P deficiency, primarily by altering their root system architecture and morphology (Zhu *et al*., 2006; Niu *et al*., 2013). For instance, they can stimulate primary and lateral root growth (Gaume *et al*., 2001; Zhu *et al*., 2005), intensify root hair development (Bates and Lynch, 2001) and root hair length and density, as reported in wheat and *Arabidopsis thaliana* (Wang *et al*., 2016; Dharmateja *et al*., 2021), and initiate cluster root formation (Wasaki *et al*., 2003; Abdolzadeh *et al*., 2010; Péret *et al*., 2011). Among the proposed root architectural models, the ‘topsoil foraging’ root ideotype has been recognized for its ability to enhance P uptake from shallow soil layers, where this element is most concentrated (White *et al*., 2013). Variations to counteract P deficiency are reported to be the production of axial roots with a shallower angle, as this could allow plants to exploit P present in the topsoil, where it is generally more available, and variations in root length, root diameter and surface area (da Silva *et al*., 2016; Campos *et al*., 2018). It is worth noting that distinct genotypic responses in root morphology to P limitations have been documented in various crops, including wheat (James *et al*., 2016; Nguyen and Stangoulis, 2019). It has been reported that some wheat genotypes can withstand P deficiency, while others are markedly affected (Deng *et al*., 2018). This differential P utilization offers an intriguing study area, opening new possibilities for developing wheat varieties that are resilient under low P conditions, thereby contributing to sustainable agricultural practices.

Several factors linked to roots are known to modulate PUE, including root hair properties (Bates and Lynch, 2001; Gahoonia and Nielsen, 2004), kinetic parameters of uptake and changes in root-induced pH levels (Gahoonia and Nielsen, 1992), root exudation processes (Marschner *et al*., 2011) and soil moisture conditions (Gahoonia *et al*., 1994). In addition, in the field, association of roots with soil microbes able to solubilize P may also affect and promote P acquisition by crops (Marschner *et al*., 2011). All these factors, representing reactions to low P conditions, can relate to different strategies, either linked to morphological or functional root traits. A trade-off between root morphology (P scavenging) and exudation (P mobilization) has been reported in a set of *Triticum aestivum* genotypes (Wang *et al*., 2023). Similarly, it has been reported that ancient maize genotypes may outperform modern cultivars under low P conditions due to stronger exudation despite a less developed root system (Li *et al*., 2019). Given these complex relationships, differences in soil P acquisition across genotypes have to be assessed under well characterized abiotic and biotic conditions, using tools able to screen root morphology variations. Different non-invasive root screening systems of plants during their growth have started to be established in recent years (Atkinson *et al*., 2019). Among them, Rhizotubes (Jeudy *et al*., 2016) are now recognized as key tools for studying root phenotypes (Maslard *et al*., 2021). The interest in exploring root traits is indeed increasing, particularly in the context of enhancing the adaptability and productivity of wheat under climate change scenarios, thanks to advances in high-throughput phenotyping and genotyping (Nirmalaruban *et al*., 2024). However, assessing nutrient use efficiency in plants is complicated by the different metrics and definitions used across studies. For nitrogen indices, for example, issues of inconsistent metrics across different crops have been highlighted (Weih *et al*., 2018). Regarding P indices, a recent study on *Vigna radiata* evaluated diverse selection traits to identify P-efficient genotypes, highlighting the complexity of PUE as a multifaceted trait (Reddy *et al*., 2021). Similarly, a study on *Coffea* spp. cultivars assessing P uptake and use efficiencies under varying soil P availabilities showed the complexity of using different metrics (Neto *et al*., 2016). Despite advances in phenotyping, the lack of a clear, quantifiable trait linked to P deficiency tolerance limits the efficiency of screening for P-efficient genotypes, as no single marker has yet emerged as an early synthetic indicator of P efficiency.

Here, a trial with 26 different bread wheat genotypes was realized at two contrasted P levels at two distinct phenotyping platforms, i.e. ALSIA (Metaponto, Italy) and 4PMI (Dijon, France) using Rhizotubes. The PAE and PUE of these genotypes in response to varying P levels was characterized. This trial was pivotal in establishing a root phenotyping flowchart to evaluate bread wheat response to varying P levels at the genotypic level, identifying key root traits for pre-breeding studies, as well as defining synthetic useful indicators with agronomic relevance to be used by agronomists and breeders in field conditions. Our results highlight genotypic-specific root-driven adaptation to low P and support multitrait selection to improve P efficiency in wheat breeding.

## MATERIALS AND METHODS

### Plant growth conditions

Twenty-six genotypes of *Triticum aestivum* were used for the study (Table 1). In detail, among the assessed genotypes, three were developed by RAGT Seeds Ltd (UK) and five by the International Maize and Wheat Improvement Center (CIMMYT, Mexico). All tested genotypes are winter wheat cultivars, with the exception of Calixo (Köhl *et al*., 2019) and CIMMYT genotypes, which are spring wheat cultivars. Two types of cylindrical Rhizotubes were used, both enabling dynamic root phenotyping. The ones used at the 4PMI platform (Dijon, France; Jeudy *et al*., 2016) feature separate zones for root growth, substrate and nutrients, thanks to a membrane that permits the flow of nutrients, and allows rhizodeposits to reach roots but prevents them crossing the membrane. The plexiglass-made Rhizotubes used at the ALSIA platform (Italy) consisted of transparent containers with a height of 60 cm and a diameter of 10 cm. This design allows monitoring of the root system as it develops and the transparency of the glass ensures that the entire root system is visible. The cylindrical shape, coupled with its dimensions, offers ample space for the roots to grow, ensuring that the plant is not constrained and that root growth is as natural as possible within the limits of the tubes. At 4PMI, Rhizotubes were filled with a mixture of sand and perlite (1:3, v:v), while at ALSIA the Rhizotubes were filled with 4.1 kg of soil without P. Soil substrate parameters (ALSIA) used in the experiment are reported in Table 2. Mean day/night temperatures were 23.3/19.3 °C at 4PMI and 19/15 °C at ALSIA. The relative humidity was controlled to 65 and 80 % at the 4PMI and ALSIA platforms, respectively. The photoperiod was set to 16 h thanks to artificial lighting at 4PMI (PAR of 355 μmol m^−2^ s^−1^) supplied with sodium lamps (400 W lamp, HPS Plantastar, Osram, Munich, Germany). At ALSIA, the average daily PAR was 10 000 000 μmol m^−2^ d^−1^ (minimum 720 000 and maximum 20 000 000).

**Table 1.** List of the tested bread wheat genotypes.

Genotype	Seed company	Code ID
Advisor	Limagrain Europe	UN 7125-3
Alessio	Lemaire Deffontaines	SZD 0989A
Annecy	Lemaire Deffontaines	LD 6009
Armada	Limagrain Europe	NSA 07-5274
Bagou	Saaten Union	SUR 98504-20
Calixo	Secobra	SEC 426-01-2 b
CIMMYT4	CIMMYT	NA
CIMMYT10	CIMMYT	NA
CIMMYT11	CIMMYT	NA
CIMMYT12	CIMMYT	NA
CIMMYT15	CIMMYT	NA
Complice	Florimond Desprez	FD 12186
Crusoe	LG	NA WW 25
Donator	Unisigma	UN 9245-5
Foxyl	KWS Momont	MH 12-29
Gedser	Sem Partners	NOS 13011.21
Hereward	Florimond Desprez	CWW 87-2
Hyking	Saaten Union	SURH 4379-380
Johnson	Saaten Union	SUR 260-83
Ionesco	Secobra	SC 36195 JT 21
Pibrac	Syngenta	SY 113007
RGT Lexio	RAGT	RW 21626
RGT Libravo	RAGT	RW 21310
Robigus	KWS Momont	CPBT W78
Rubisko	RAGT	RW 20957
Soissons	Florimond Desprez	FD85058

NA, not available.

**Table 2.** Chemical–physical features of the soil substrate used in the ALSIA platform.

Parameter	Value	Unit
Bulk density	553	g L^−1^
pH	8.1	
Electrical conductivity	13	mS m^−1^
Ammonium nitrogen (N-NH_4_)	10.68	mg L^−1^
Nitrate nitrogen (N-NO_3_)	21.45	mg L^−1^
Mineral nitrogen (calculated)	32.13	mg L^−1^
Calcium	73.55	mg L^−1^
Magnesium	11.96	mg L^−1^
Potassium	16.50	mg L^−1^
Sodium	14.69	mg L^−1^
Iron	0.65	mg L^−1^
Manganese	0.09	mg L^−1^
Copper	0.02	mg L^−1^
Zinc	<0.01	mg L^−1^
Phosphorus	<0.02	mg L^−1^

At both platforms, seeds were vernalized. At 4PMI, seeds were disinfected, then imbibed at 20 °C for 12 h then vernalized at 4 °C for 24 h in a Fitoclima S600 germinator (Aralab, Rio de Mouro, Portugal). Seeds were sown on 27 March 2023 at ALSIA and on 12 June 2023 in 4PMI. The end of the trial was established on 22 June 2023 for ALSIA for a total of 96 d and on 11 July 2023 at 4PMI for a total of 28 d. Seedlings were grown in Rhizotubes allowing the visualization of the root system (Jeudy *et al*., 2016). In total, 312 Rhizotubes (26 genotypes × 6 tubes × 2 P levels) were used at 4PMI, while 208 Rhizotubes (26 genotypes × 4 tubes × 2 P levels) were used at ALSIA. At ALSIA, three seeds per tube were sown, in soil, and watered to reach optimal water-holding capacity.

At both platforms, the P treatments were carried out through the use of Hoagland solutions with different NH_4_H_2_PO_4_ concentrations. The Hoagland solution consisted of 3 mm KNO_3_, 2 mm CaCl_2_, 2 mm MgSO_4_·7H_2_O, 9.14 µm MnCl_2_·4H_2_O, 0.76 µm ZnSO_4_·7H_2_O, 0.32 µm CuSO_4_·5H_2_O, 0.49 µm NaMoO_4_·2H_2_O and 52 µm KFeEDTA. In addition, the solution was modified to create two distinct P levels: a standard level (P+), representing optimal P availability, and a low level (P−) to simulate P-deficient conditions, i.e. the concentration of NH_4_H_2_PO_4_ was 0.0032 mm for P− (corresponding to ∼1.73 kg ha^−1^) and 0.3 mm for P+ (corresponding to ∼162 kg ha^−1^). In addition, the concentration of NH_4_ was adjusted to equally supply nitrogen in the different P treatments: 0.99 mm for P− and 0.70 mm for P+. The composition of other nutrients remained constant across both levels. At 4PMI, plants were watered twice a day with 250 mL of nitrogen-free nutritive solution, while at ALSIA Hoagland solution was provided as a single treatment of 50 mL at the beginning of the trial.

### Root scanning and image analysis

In both platforms, Rhizotubes were coupled with dedicated high-resolution cameras, allowing us to observe root growth over time and to assess differences in growth kinetics under different nutritional conditions. At 4PMI, each Rhizotube was imaged three times a week using the high-throughput aerial and root phenotyping booths in the 4PMI RhizoCab^®^ HD (Jeudy *et al*., 2016). At ALSIA, root phenotyping scanning was performed by means of two measurements each week.

At the 4PMI platform 4546 high-resolution images (12 000 × 12 000 pixels) of the growth kinetics of wheat root development were analysed in two main steps. First, a raw processing step in order to separate the pixels of the root system from the background was obtained using a deep learning semantic segmentation model. The output binary image was cleaned of its artefacts, keeping the biggest continuous white pixels from the top, which allowed the removal of noise and false detection. The second step consisted in getting some measures, based on OpenCV and Scikit-image libraries. Conversions from pixels to mm, mm^2^ and mm^3^ were done using the coefficient value of 0.0042, corresponding to 600 dpi image resolution. The projected area is the sum of the white pixels in the binary image.

The plant phenotyping facility at ALSIA uses customized software algorithms to evaluate the growth of plant root systems. The facility is equipped with rotating cylindrical rhizotrons, which facilitates multiple imaging perspectives of the roots. By taking multiple shots at various angles, a holistic view of the root system is ensured. Individual captures (six images at 60° intervals) are merged, resulting in a panorama image that offers a 360° perspective of the root layout, using rotation correction techniques (Renò *et al*., 2021). The singular value decomposition (SVD) algorithm is used to attenuate noise by focusing on the lower rank approximation of the panorama, ensuring high resolution of the resulting images. Through automatic segmentation, images of roots are isolated by effectively filtering out the Laplacian of Gaussian. This results in binary images where the roots are distinctly recorded and the corresponding root growth area is measured.

### Plant measurements at harvest

At ALSIA, harvest was performed 97 d after sowing while it was earlier, 28 d after sowing, at 4PMI. Destructive sampling for roots, shoots and soil was carried out at the end of the experiment. During sampling, fresh weight, plant height and the number of culms were recorded. Shoots and roots were also oven-dried at 60 °C until constant mass and weighed for biomass determination (dry weight). At ALSIA both root and shoot samples, as well as soil, were subsequently used for P analysis (performed by Demetralab s.r.l., Pescia, PT, Italy). The P quantification protocol followed a spectrophotometric method based on phosphomolybdate complex coloration. Both root and shoot tissues were homogenized before taking an aliquot for analysis. The specific amount of plant material used for digestion was 0.5 g, while 1 g was used for the extraction of plant-available P of soil samples. The digestion process was performed using 4 mL of HNO_3_ and 4 mL of H_2_O_2_ following the protocol described in Mills and Jones (1996).

At 4PMI, P contents in root and shoot at harvest were measured by ICP MS (Plateforme PLATIN’ [Plateau d’Isotopie de Normandie], Université de Caen, Normandie, Caen, France). Content of P (using ^31^P) was measured by high-resolution inductively coupled plasma mass spectrometry (HR–ICP–MS, Thermo Scientific, Element 2™, Bremen, Germany) with prior microwave acid sample digestion (Multiwave ECO, Anton Paar, les Ulis, France) using 800 µL of concentrated HNO_3_ (70 %, bidistilled with a subPUR acid distiller, Milestone), 200 µL of H_2_O_2_ (SCP Science, Quebec, Canada) and 900 µL of ultrapure water for 40 mg DW. Both soil and plant samples were spiked with two internal standard solutions of gallium and rhodium for final concentrations of 5 and 1 mg L^−1^, respectively, diluted to 50 mL with ultrapure water to obtain solutions containing 2.0 % (v/v) of nitric acid, and then filtered at 0.45 mm using a Teflon filtration system (Digifiltre, SCP Science, Villebon-sur-Yvette, France). Concentrations were expressed in parts per million (ppm). The corresponding spectrometry measurements were performed by the PLATIN’ core facility.

### Estimated indicators

At both platforms, five different P traits related to P use efficiency were calculated at harvest.

The PAE was calculated as in Han *et al*. (2022) with the following formula:


$$\mathrm{PAE}=\frac{P_{\mathrm{uptake}}}{P_{\mathrm{applied}}}\cdot 100=\frac{P\;\mathrm{per}\;\mathrm{plant}(g)}{P\;\mathrm{per}\;\mathrm{substrate}\;\mathrm{litre}(g)}\cdot 100$$

in which *P*_applied_ refers to the P provided through the Hoagland solution.

The PUE was calculated as in Syers *et al*. (2008) and Neto *et al.* (2016) by the balance method, as follows:


$$\mathrm{PUE}=\frac{\mathrm{Dry}\;\mathrm{Weight}(g)}{P\;\mathrm{concentration}\;\mathrm{in}\;\mathrm{plant}(\mathrm{mM})\cdot \mathrm{Dry}\;\mathrm{Weight}(g)}$$

The phosphorus root acquisition efficiency (PRAE) was calculated as:


$$\mathrm{PRAE}=\frac{P_{\mathrm{uptake}}(g)}{\mathrm{Root}\;\mathrm{Biomass}(g)}$$

In addition, agronomic P use efficiency (APUE) was calculated as in Neto *et al*., 2016 (adapted from Oliveira *et al*., 1987) as follows:


$$\mathrm{APUE}=\frac{\mathrm{DW}\;\mathrm{of}\;P+-\mathrm{DW}\;\mathrm{of}\;P-}{\mathrm{Total}\;\mathrm{available}\;P+-\mathrm{Total}\;\mathrm{available}\;P-}$$

The P relative efficiency as a percentage of the ratio between biomasses produced at low P level (P−) versus high P level (P+) was calculated with the following formula:


$$P\_\mathrm{Relative}\;\mathrm{Efficiency}=\frac{\mathrm{Plant}\;\mathrm{Biomass}\;P-}{\mathrm{Plant}\;\mathrm{Biomass}\;P+}\cdot 100$$

Finally, in order to obtain a single synthetic indicator (namely ‘P indicator’) that included all five of the above-mentioned variables for each of the 26 genotypes, data were normalized in a range between 0 and 1 and summed.

### Statistical analyses

The experimental design was a randomized complete block design. Data were analysed using two-way ANOVA with interaction, with genotypes as the first factor and P level as the second one (*P* < 0.05). Prior to performing ANOVA, data were assessed for homogeneity of variance using the Levene test and for normality using the Shapiro–Wilk test. Pairwise comparisons within each genotype to assess differences in P levels, when ANOVA for this factor was significant, was performed by Student’s *t*-test with Bonferroni correction (*P* < 0.05). Root phenotyping data from ALSIA (root surface area) were analysed by fitting, for each genotype and P level, a mixed-effects model using the lme4 R package (Bates *et al*., 2015). Linear mixed-effects models were constructed to assess the impacts of genotype, treatment and their interaction with the temporal growth curve. The model accounted for B-spline transformation of the time variable to model the growth trajectory over time; genotype and P level were treated as fixed effects. Plant position, identified by specific barcodes, was included as a random effect to account for within-plant variability and limit type I errors due to non-independence of observations. The lmerTest R package (Kuznetsova *et al*., 2017) was used to assess the significance of differences for the fixed effects in the mixed models by ANOVA with the Kenward–Roger approximation for degrees of freedom. Finally, to assess the relationships between PAE, PUE and PRUE under both P+ and P− conditions, a Pearson correlation analysis was conducted (*P* < 0.05).

### Comparison between outcomes from the two different platforms

The P indicator values of the wheat genotypes from the two distinct experimental platforms, i.e. ALSIA and 4PMI, were compared through three different approaches. First, a Pearson correlation coefficient was used to measure the linear correlation between the datasets from ALSIA and 4PMI. Second, to assess the agreement between the two estimated P indicator values for each genotype, the Lin concordance correlation coefficient (CCC) was used (Lin *et al.*, 2002). Third, Bland–Altman plot analysis was also applied to further investigate the agreement between the two platforms. This method involves plotting the differences between two measurements against their averages, providing a visual assessment of agreement. The plot utilized statistical limits of agreement based on the mean and standard deviation of the differences (Dewitte *et al*., 2002). In addition, the PAE, PRAE, PUE and APUE values from the two platforms were compared through a Pearson correlation analysis.

## RESULTS

**Fig. 1.**
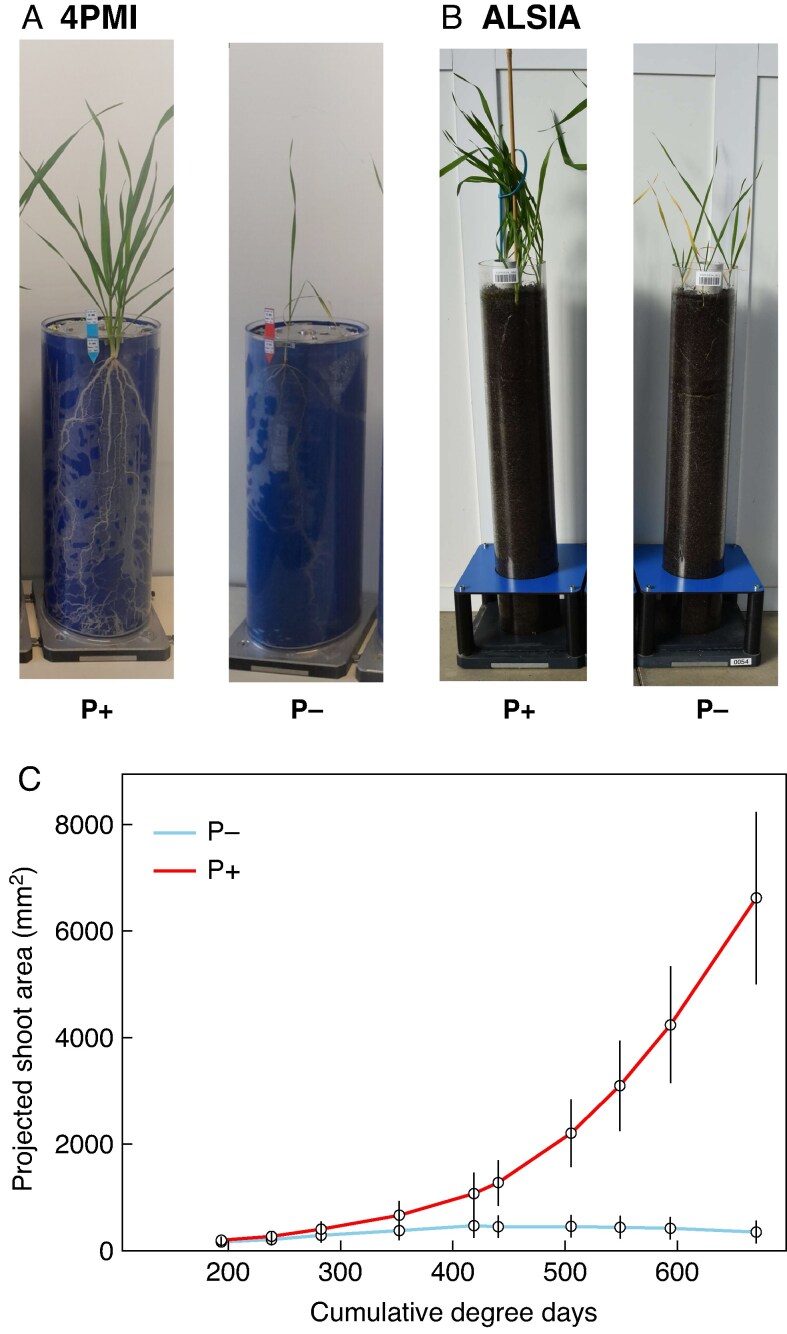
Representative examples of wheat genotypes grown at the ALSIA and 4PMI facilities. (A, B) Genotype Bagou under P+ and P− conditions at 4PMI (A) and ALSIA (B). (C) Evolution of the projected area of shoot for Bagou under P+ and P−.

A representative picture of plants of genotype Bagou grown on Rhizotubes is reported in Fig. 1, and a short video of segmented shoot and root images of the same genotype under P+ and P− from the 4PMI platform is reported as Supplementary Data Video S1. In order to assess how the different genotypes reacted to the P treatment, integrative variables related to P use efficiency were calculated. The P use efficiency was expressed in PAE and PUE. PAE depends upon root system architecture and its various phenes (York *et al*., 2013), especially those that keep more roots in the upper soil layers (Hammond *et al*., 2009; George *et al*., 2011; Lynch, 2011, 2019; White *et al*., 2013; Jia *et al*., 2018; Sun *et al*., 2018); PUE reflects how plants produce their biomass from the acquired P and is mostly related to P use for cellular processes (e.g. DNA), protein build-up and energy supply. On the other hand, PRAE is focused on the efficiency of the compartment responsible for P acquisition (i.e. root) in acquiring P linked to root architecture (typology, prospection etc.), biomass, and functions.

### Phosphorus acquisition efficiency

Under P+ conditions at ALSIA, the genotype Rubisko showed the highest PAE (80.34), followed by Alessio (62.53) and RGT Libravo (59.49), while at 4PMI Gedser (44.28), RGT Libravo (44.06) and Soissons (41.01), ranked the highest. Conversely, at ALSIA the genotypes with the lowest PAE under P+, suggesting a less efficient conversion of absorbed P into biomass conditions, were Robigus (15.88), Donator (20.22) and CIMMYT11 (19.79), and at 4PMI they were CIMMYT12 (20.17) and CIMMYT10 (26.6) (Table 3).

**Table 3.** PAE, PRAE, PUE, P relative efficiency and APUE calculated on data from large-scale trials performed at 4PMI (France) and ALSIA (Italy). For PAE, PRAE and PUE values, mean values (± standard deviation) are reported (4PMI *n*= 6; ALSIA n = 4).

Genotype	4PMI								ALSIA							
	PAE (P−)	PRAE (P−)	PUE (P−)	PAE (P+)	PRAE (P+)	PUE (P+)	P relative efficiency	APUE	PAE (P−)	PRAE (P−)	PUE (P−)	PAE (P+)	PRAE (P+)	PUE (P+)	P relative efficiency	APUE
Advisor	57.86 (±3.48)	0.00147 (±0.00018)	37.54 (±2.53)	36.79 (±9.72)	0.02963 (±0.00205)	181.86 (±75.69)	15.2	40.96	20.75 (±2.45)	0.004 (±0.00034)	24.10 (±4.77)	35.07 (±1.90)	0.063 (±0.00421)	165.28 (±32.71)	47.69	59.8
Alessio	68.35 (±9.26)	0.00157 (±0.00015)	54.04 (±8.90)	30.17 (±7.18)	0.02879 (±0.00187)	144.90 (±69.75)	20.8	32.56	29.68 (±2.12)	0.006 (±0.00035)	89.95 (±18.01)	62.53 (±3.29)	0.059 (±0.00363)	92.68 (±18.56)	63.34	66.8
Annecy	55.23 (±7.75)	0.00167 (±0.00019)	32.85 (±16.69)	28.45 (±2.73)	0.03123 (±0.00064)	77.40 (±20.85)	20.0	26.83	20.87 (±0.69)	0.006 (±0.00081)	29.89 (±1.61)	36.79 (±3.76)	0.065 (±0.00840)	157.53 (±8.49)	66.15	51.6
Armada	62.15 (±5.55)	0.00145 (±0.00015)	57.34 (±17.38)	27.88 (±6.35)	0.02584 (±0.00262)	158.15 (±38.17)	19.7	34.59	22.39 (±2.26)	0.006 (±0.00022)	25.73 (±2.51)	35.53 (±0.96)	0.065 (±0.00223)	163.14 (±15.94)	73.93	35.7
Bagou	46.40 (±14.68)	0.00148 (±0.00016)	26.51 (±18.07)	33.88 (±8.41)	0.03278 (±0.00398)	134.27 (±80.06)	13.7	35.96	18.11 (±2.91)	0.006 (±0.00178)	38.49 (±3.76)	51.64 (±12.24)	0.075 (±0.02260)	112.23 (±10.96)	40.81	91.3
Calixo	56.25 (±11.28)	0.00237 (±0.00008)	29.48 (±4.26)	30.44 (±1.34)	0.03618 (±0.00162)	144.64 (±23.07)	15.6	35.78	19.77 (±0.98)	0.011 (±0.00166)	20.99 (±2.82)	24.74 (±1.34)	0.072 (±0.01098)	234.25 (±31.51)	79.73	44.5
CIMMYT4	70.29 (±14.19)	0.00182 (±0.00012)	51.40 (±15.59)	29.03 (±4.25)	0.03109 (±0.00198)	122.03 (±42.69)	17.9	30.59	24.88 (±2.91)	0.010 (±0.00089)	21.55 (±4.29)	29.13 (±1.39)	0.08097 (±0.00719)	198.94 (±39.60)	97.56	5.9
CIMMYT10	88.39 (±9.91)	0.00222 (±0.00016)	45.80 (±11.32)	26.60 (±2.33)	0.03482 (±0.00441)	77.23 (±16.83)	21.9	23.19	19.38 (±1.55)	0.006 (±0.00076)	24.45 (±3.07)	22.93 (±1.98)	0.083 (±0.01045)	252.77 (±31.73)	77.6	48.0
CIMMYT11	56.10 (±11.28)	0.00185 (±0.00017)	28.56 (±10.31)	30.64 (±1.93)	0.03084 (±0.00121)	100.09 (±29.40)	27.2	30.01	14.10 (±0.77)	0.008 (±0.00119)	27.36 (±3.31)	19.79 (±1.35)	0.100 (±0.01478)	292.88 (±35.48)	93.47	11.1
CIMMYT12	73.46 (±14.45)	0.00180 (±0.00023)	40.16 (±14.60)	20.17 (±8.46)	0.0268 (±0.00294)	66.03 (±48.33)	18.6	19.00	16.34 (±1.85)	0.009 (±0.00078)	28.42 (±4.16)	22.21 (±1.65)	0.111 (±0.00858)	260.89 (±38.23)	78.07	43.9
CIMMYT15	62.71 (±10.61)	0.00152 (±0.00013)	49.68 (±3.92)	33.65 (±7.82)	0.02437 (±0.00387)	175.23 (±69.46)	13.8	37.93	18.07 (±0.84)	0.008 (±0.00061)	27.42 (±3.58)	36.15 (±1.82)	0.080 (±0.00635)	160.31 (±20.94)	61.67	94.3
Complice	76.70 (±19.24)	0.00188 (±0.00020)	50.53 (±23.30)	33.43 (±4.39)	0.03288 (±0.00342)	148.26 (±18.02)	19.5	35.68	21.08 (±2.39)	0.005 (±0.00121)	21.83 (±2.71)	29.78 (±4.48)	0.066 (±0.01332)	194.64 (±24.18)	63.5	56.2
Crusoe	53.62 (±7.47)	0.00171 (±0.00014)	32.21 (±13.28)	36.39 (±5.69)	0.03297 (±0.00243)	179.03 (±68.00)	13.6	41.54	13.50 (±0.91)	0.005 (±0.00031)	18.03 (±3.43)	22.88 (±0.98)	0.071 (±0.00372)	253.28 (±48.20)	69.84	22.3
Donator	41.50 (±5.15)	0.00140 (±0.00020)	30.87 (±4.15)	32.16 (±4.86)	0.03108 (±0.00311)	104.44 (±32.79)	16.8	31.92	10.61 (±0.40)	0.006 (±0.00038)	16.25 (±2.79)	20.22 (±0.93)	0.078 (±0.00485)	286.63 (±49.22)	51.63	43.3
Foxyl	59.09 (±3.99)	0.00150 (±0.00006)	37.24 (±7.91)	35.74 (±4.18)	0.02887 (±0.00066)	154.74 (±45.10)	15.4	38.87	13.86 (±0.59)	0.007 (±0.00107)	29.79 (±4.51)	25.09 (±3.02)	0.073 (±0.01119)	231.02 (±34.97)	51.85	45.7
Gedser	81.12 (±10.89)	0.00170 (±0.00026)	55.38 (±17.91)	44.28 (±4.38)	0.03072 (±0.00082)	202.72 (±39.38)	16.8	45.12	21.85 (±1.45)	0.006 (±0.00042)	32.28 (±1.95)	40.08 (±2.11)	0.070 (±0.00506)	144.60 (±8.73)	41	97.2
Hereward	58.35 (±13.05)	0.00163 (±0.00004)	37.28 (±9.28)	36.36 (±3.05)	0.02882 (±0.00064)	187.82 (±36.89)	14.6	42.08	16.89 (±1.77)	0.005 (±0.00047)	19.37 (±5.54)	22.89 (±1.71)	0.006 (±0.00056)	200.85 (±57.49)	73.44	29.9
Hyking	66.22 (±18.55)	0.00169 (±0.00005)	46.12 (±22.94)	35.37 (±3.41)	0.02966 (±0.00375)	184.86 (±52.50)	16.2	41.36	50.34 (±2.98)	0.006 (±0.00053)	58.35 (±3.70)	48.03 (±3.73)	0.057 (±0.00530)	120.67 (±7.65)	94.19	8.8
Ionesco	62.72 (±16.04)	0.00176 (±0.00012)	40.40 (±16.64)	38.34 (±6.14)	0.02929 (±0.00311)	207.57 (±65.30)	14.5	44.3	31.15 (±2.96)	0.005 (±0.00039)	26.22 (±1.74)	35.22 (±2.27)	0.063 (±0.00491)	164.55 (±10.94)	44.93	66.8
Johnson	53.39 (±11.07)	0.00122 (±0.00017)	43.99 (±14.38)	28.41 (±2.81)	0.02994 (±0.00253)	113.54 (±14.49)	19.8	29.74	23.49 (±3.84)	0.006 (±0.00043)	22.10 (±3.33)	26.70 (±1.65)	0.052 (±0.00362)	217.06 (±32.71)	94.69	3.5
Pibrac	68.81 (±11.52)	0.00142 (±0.00006)	53.12 (±10.50)	34.62 (±5.63)	0.03241 (±0.00439)	138.48 (±36.04)	20.4	34.68	27.83 (±2.16)	0.005 (±0.00062)	25.23 (±5.58)	38.51 (±3.14)	0.070 (±0.00710)	150.48 (±33.27)	68.28	0.6
RGT Lexio	61.81 (±12.60)	0.00158 (±0.00010)	48.89 (±17.98)	35.34 (±7.50)	0.03268 (±0.00575)	176.10 (±47.01)	16.5	42.27	18.19 (±0.92)	0.006 (±0.00029)	23.35 (±1.10)	30.50 (±1.39)	0.061 (±0.00284)	190.03 (±8.97)	73.49	33.4
RGT Libravo	59.25 (±2.29)	0.00173 (±0.00016)	35.43 (±9.44)	44.06 (±3.59)	0.03561 (±0.00147)	146.12 (±89.66)	17.0	35.17	28.43 (±2.30)	0.006 (±0.00034)	83.57 (±4.40)	59.49 (±2.40)	0.058 (±0.00334)	97.43 (±5.13)	57.29	72.1
Robigus	48.27 (±10.44)	0.00165 (±0.00021)	25.79 (±11.17)	34.91 (±6.74)	0.0282 (±0.00268)	176.45 (±64.80)	12.4	41.33	13.82 (±1.69)	0.007 (±0.00071)	15.14 (±1.32)	15.88 (±1.14)	0.077 (±0.00762)	364.95 (±31.90)	88.41	9.4
Rubisko	50.95 (±10.14)	0.00143 (±0.00014)	37.87 (±14.53)	31.22 (±4.80)	0.0292 (±0.00130)	140.47 (±38.63)	16.7	35.02	14.28 (±1.11)	0.007 (±0.00234)	51.22 (±12.26)	80.34 (±21.11)	0.091 (±0.03029)	72.14 (±17.27)	36.95	98.0
Soissons	68.06 (±13.82)	0.00153 (±0.00003)	67.33 (±27.78)	41.01 (±3.94)	0.02983 (±0.00097)	263.52 (±73.75)	16.1	48	30.31 (±4.51)	0.006 (±0.00072)	27.59 (±1.59)	34.25 (±3.27)	0.061 (±0.00709)	169.23 (±9.75)	84.36	29.3

Under P− conditions, at ALSIA genotypes with the lowest PAE were Donator (10.61), Robigus (13.82) and Crusoe (13.49), while the highest values were observed in Hyking (50.34), Ionesco (31.15) and Soissons (30.31). At 4PMI under similar P− conditions, Donator (41.50), Bagou (46.40) and Robigus (48.27) were the least efficient while Complice (76.7), Gedser (81.12) and CIMMYT10 (88.39) had the highest PAE (Table 3).

### Phosphorus utilization efficiency

Under P+ conditions, at ALSIA Robigus showed the highest PUE of 364.95 followed by CIMMYT11 (292.88) and Donator (286.63). At the lower end, Rubisko (72.14), Alessio (92.68) and RGT Libravo (97.43) had the lowest PUE values. At 4PMI, Gedser (202.72), Ionesco (207.57) and Soissons (263.52) had the highest PUE, and CIMMYT12 (66.03), CIMMYT10 (77.23) and Annecy (77.4) displayed the lowest PUE (Table 3).

Under P− conditions, the top-performing genotypes were different, with Alessio showing the highest PUE of 89.95, followed by RGT Libravo (83.57) and Hyking (58.35), while the lowest PUE values were observed in Robigus (15.14), Donator (16.25) and Crusoe (18.03). At 4PMI, Gedser (55.38), Armada (57.34) and Soissons (67.33) were the top performers and Robigus (25.79) and Bagou (26.51) displayed the lowest PUE (Table 3).

### Phosphorus root acquisition efficiency

In ALSIA under P+, the lowest PRAE values were recorded for Hereward (0.00625), followed by Johnson (0.05159) and Hyking (0.05783). At 4PMI, the lowest values were observed for CIMMYT15 (0.02437), Armada (0.02584) and CIMMYT12 (0.0268). Conversely, the highest PRAE values at ALSIA were seen with CIMMYT11 (0.11142) and CIMMYT10 (0.10042), while at 4PMI RGT Libravo (0.03561) and Calixo (0.03618) had the highest PRAE values (Table 3).

In ALSIA under P−, the lowest PRAE values were associated with Ionesco (0.00521), Advisor (0.00527) and Hereward (0.00544), while at 4PMI the lowest PRAE values were detected for Johnson (0.00122), Donator (0.00140) and RGT Lexio (0.00142). Highest PRAE values were associated with Calixo (0.01077) and CIMMYT12 (0.00983) at ALSIA, while at 4PMI they were associated with Calixo (0.00237) and CIMMYT10 (0.00222) (Table 3).

**Fig. 2.**
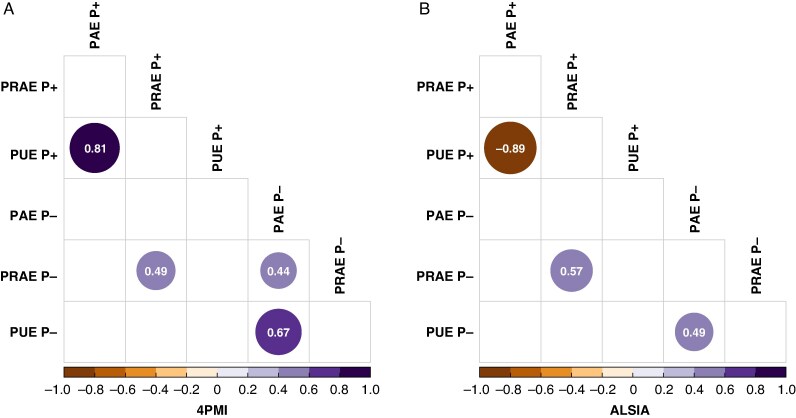
Correlation matrices of PAE, PRAE and PUE at two P levels (P+ and P−) at the different platforms, 4PMI (A) and ALSIA (B). The matrices show the correlation coefficients between each pair of indices. Size and colour of circles are proportional to the Pearson correlation. Only significant (*P* < 0.05) coefficients are plotted.

The Pearson correlation analysis allowed us to obtain the relationships between PAE, PUE and PRAE under P+ and P− conditions (Fig. 2). Under P+ conditions, a significant positive correlation (*r* = 0.81, *P* < 0.001) was observed between PAE and PUE at 4PMI, while at ALSIA a significant negative correlation was observed (*r* = −0.89, *P* < 0.001). Under P− conditions, a positive correlation between PAE and PUE was observed at both 4PMI and ALSIA (*r* = 0.67 and 0.49, respectively, *P* < 0.05). The correlation between PAE and PRAE was significant under the P− condition in 4PMI data exclusively (*r* = 0.44, *P* < 0.05; Fig. 2).

### Phosphorus relative efficiency

The decrease in biomass production noticed on lowering P availability was calculated as a way to measure the plasticity of plants produced under P deficiency versus optimal P nutrition. At ALSIA, genotypes CIMMYT4, Johnson and CIMMYT11 appeared to be less sensitive to P deprivation in terms of biomass loss, with values >90. The genotypes Rubisko, Bagou and Gedser were the most affected, with reported efficiency values of 36.95, 40.81 and 41.00, respectively. At 4PMI, the CIMMYT12 and CIMMYT10 genotypes were the least affected, showing high values of P relative efficiency, while Robigus was the most affected by P deficiency according to this index (Table 3).

### Agronomic P use efficiency

**Fig. 3.**
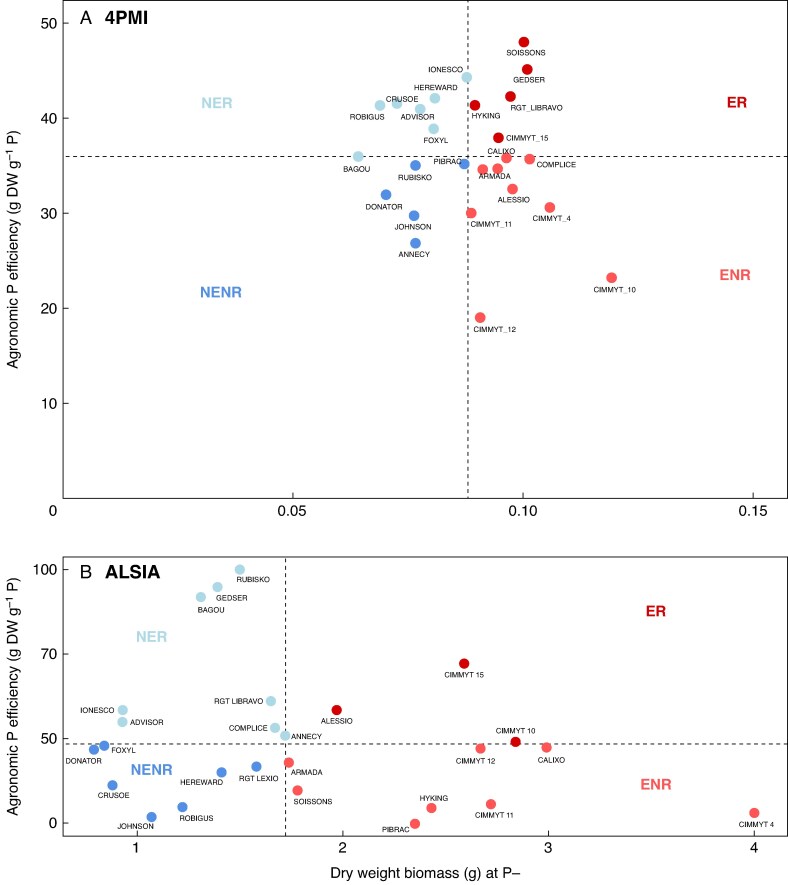
Relationship between plant dry matter (DW) at P− and responsiveness to P, measured as APUE, for the different bread wheat genotypes at (A) 4PMI and (B) ALSIA. NER, non-efficient and responsive; ER, efficient and responsive; ENR, efficient and non-responsive; NENR, non-efficient and non-responsive.

The APUE values are reported in Table 3. At ALSIA, Rubisko demonstrated the highest APUE values (98), followed by Gedser (97.2) and Bagou (91.3), whereas at 4PMI these were Soissons (48.00), Gedser (45.12) and Ionesco (44.30). Conversely, at ALSIA, genotypes with the lowest APUE were Pibrac (0.60), Johnson (3.50) and CIMMYT4 (5.90), while CIMMYT12 (19.00), CIMMYT10 (23.19) and Annecy (26.83) ranked the lowest APUE at 4PMI (Table 3). Based on APUE values and dry weight of biomass of whole plants (roots and shoots), the genotypes were categorized into four groups, i.e. efficient and responsive (ER), efficient and non-responsive (ENR), non-efficient and responsive (NER) and non-efficient and non-responsive (NENR), as in Neto *et al*. (2016). The distribution of genotypes in these four groups is reported in Fig. 3.

At ALSIA the ENR group comprised eight genotypes, the ER group three genotypes, the NER group eight genotypes, and the NENR group seven genotypes (Fig. 3). Classification based on APUE values allowed us to identify Alessio, CIMMYT10 and CIMMYT15 as the ER genotypes (Fig. 3). The other CIMMYT genotypes fell in the ENR group. The NENR sector included seven genotypes, such as Johnson, Crusoe and Robigus (Fig. 3).

At 4PMI the ENR group comprised nine genotypes, the ER group five genotypes, the NER group seven genotypes and the NENR group five genotypes (Fig. 3). Classification based on APUE values allowed to identify CIMMYT15, Gedser, RGT_Libravo, Hyking and Soissons as the ER genotypes (Fig. 3). As for ALSIA, the other CIMMYT genotypes fell in the ENR group. In the NENR group, genotypes such as Johnson, Donator and Annecy were included (Fig. 3).

### Shoot and root biomass

**Fig. 4.**
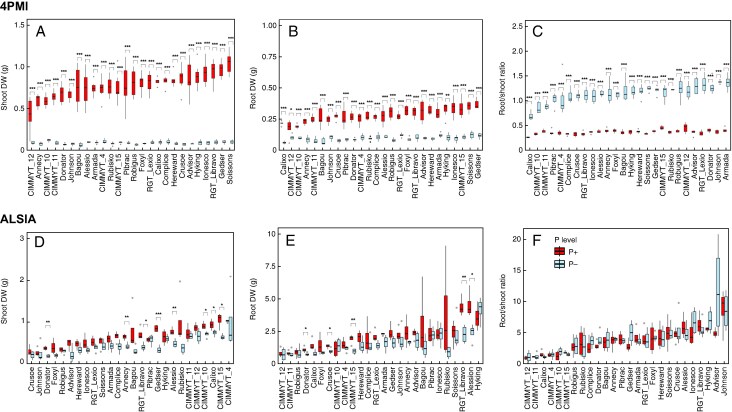
Shoot and root dry weight (DW) and root/shoot ratios under P+ and P− conditions across the studied wheat genotypes at the 4PMI (A–C; *n* = 6) and ALSIA (D–F; *n* = 4) platforms.

In order to understand from which morphological or functional traits such efficiency variation could arise, the dry weights of shoots and roots were measured to assess how the different genotypes responded to the two P levels (Fig. 4). According to ANOVA, considering 4PMI data, the factor genotype influenced dry weight of shoots (*F* = 4.916, *P* < 0.001), as well as the factor P level (*F* = 3748.612, *P* < 0.001) and the interaction between these two factors (*F* = 5.124, *P* < 0.001). Considering the dry weight of shoots measured at the ALSIA platform, the factor genotype showed a significant effect (*F* = 9.008, *P* < 0.001), as well as the P level factor (*F* = 78.091, *P* < 0.001). However, the interaction effect between genotype and P level on the dry weight of shoots was not statistically significant (*F* = 1.395, *P* = 0.0987). At both platforms, and particularly 4PMI, whatever the genotype, there was a strong effect of P treatment on growth and development and some genotypes showed a large reduction in shoot biomass when subjected to the low P level. At 4PMI, Gedser and Soissons followed by RGT_Libravo and Ionesco displayed the strongest reduction (*t*-test, *P* < 0.01) in shoot biomass when subjected to P deficiency (*t*-test, *P* < 0.05; Fig. 4). Pairwise *t*-tests within each genotype between the two P levels revealed significant differences across all comparisons with 4PMI data (Fig. 4). At ALSIA, particularly Gedser, Alessio, Annecy and Donator showed a significant strong reduction (*t*-test, *P* < 0.01) in shoot biomass under P− compared with P+. In addition, also CIMMYT15, Calixo, CIMMYT10 and RGT_Libravo showed a decrease in biomass under P− conditions compared with P+ (*t*-test, *P* < 0.05; Fig. 4).

Data on root dry weight of plants showed a similar scenario for 4PMI (Fig. 4). Significant differences in root biomass were detected in the 4PMI dataset for all of the genotypes, and ANOVA showed that both genotype and P level had a significant effect (genotype, *F* = 6.752, *P* < 0.001; P level, *F* = 2025.536, *P* < 0.001), as well as their interaction (*F* = 4.370, *P* < 0.001). As for shoot biomass, pairwise *t*-tests within genotypes between the two P levels showed significant differences (Fig. 3). Again, Gedser, Soissons, CIMMYT15 and Ionesco were strongly affected by the decrease in P supply (Fig. 4).

Regarding the ALSIA dataset, significant variation was observed among the different genotypes (*F* = 6.223, *P* < 0.001), P level (*F* = 30.314, *P* < 0.001) and the interaction between genotype and P level (*F* = 1.675, *P* = 0.0228), suggesting that the effect of genotype on root dry weight varies across different levels of P, or vice versa. The genotype Hyking showed the highest root biomass in both P levels, while most of the CIMMYT genotypes resulted in reduced root biomass under the P− level (Fig. 3). According to the *t*-test, CIMMYT15 showed a relevant reduction in root biomass under the P− condition, along with RGT_Libravo (*t*-test, *P* < 0.01). Additionally, genotypes Alessio, Crusoe and Donator also showed significant differences in root dry weight between the two P levels (Fig. 4). Concerning the root/shoot ratio, while all tested factors significantly influenced this parameter for the 4PMi dataset (*P* < 0.001), for the ALSIA dataset ANOVA highlighted a significant effect of genotype (*F* = 9.264, *P* < 2^e−16^), but no significant effect of P level and the interaction between the two variables (*F* = 3.652, *P* = 0.0578, and *F* = 1.318, *P* = 0.1570, respectively). All CIMMYT genotypes showed lower root/shoot ratios at both P levels compared with the other ones. The highest root/shoot ratios were determined in genotypes Johnson, Advisor and Hyking (Fig. 4). The P content measured in plant tissues across both 4PMI and ALSIA platforms showed a consistent reduction under P− conditions, with genotype-dependent variation (Supplementary Data Fig. S1). The observed differences in P concentration between the two platforms, higher in ALSIA, could be attributed to a dilution effect, as plants grown at ALSIA were larger at 96 d compared with those at 4PMI harvested at 28 d.

### Synthetic indicator for agronomic relevance and comparison between platforms

**Fig. 5.**
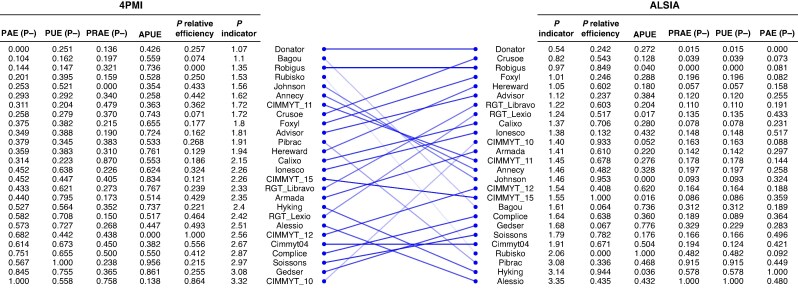
Summary tables showing genotype rankings based on normalization of the five assessed metrics under P+ and P− conditions at the 4PMI and ALSIA platforms.

**Fig. 6.**
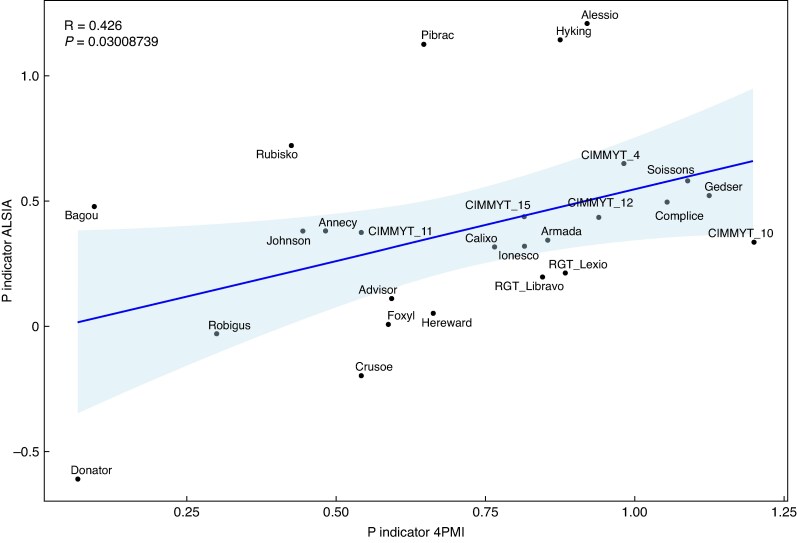
Correlation plot between P indicator values obtained at platforms 4PMI and ALSIA. The blue line represents the linear regression line with confidence intervals shaded.

Due to the complexity of managing the five relevant indicators, these were arbitrarily normalized to get a global mark between 0, demonstrating the lowest overall efficiency, and 5, representing the best-performing genotype under P deficiency (Fig. 5). This normalized indicator was termed the ‘P indicator’. According to the platform where the trial was made, the ranking differed. Among the genotypes, ∼30 % were similarly ranked (Fig. 5). A significant Pearson correlation between ALSIA and 4PMI P indicator values was detected (*r* = 0.426, *P* = 0.03; Fig. 6). Additionally, the CCC was also significant (*r* = 0.424, *P =* 0.0069). The Bland–Altman plot clearly showed that only the Pibrac genotype was outside the range of the upper and lower limits of agreement (Supplementary Data Fig. S2), indicating a high level of concordance between the indexes assessed on both platforms. On the other hand, no significant correlations between PAE, PUE, PRAE and APUE values from the two platforms were detected, with the exception of PRAE values under P−, which showed a slightly positive significant correlation (Supplementary Data Fig. S3).

### Root phenotyping data

**Fig. 7.**
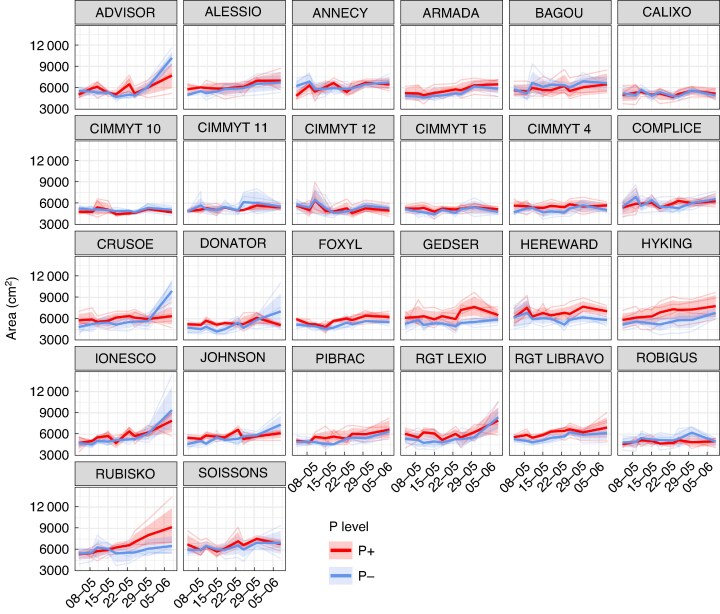
Root phenotyping data (ALSIA) from large-scale trial for P+ and P−. Coloured lines (red for P+, blue for P−) represent the fitted model growth curves for the four replicates within each genotype over time.

At ALSIA, root phenotyping data showed that, under the P− level, genotypes Advisor, Crusoe, Donator, Ionesco and RGT Lexio had pronounced growth rates with a consistent upward trend with an acceleration in growth during the latter stages (Fig. 7). Other genotypes, e.g. Rubisko, showed the same increasing trend in P+ exclusively. Statistical assessment of differences between fitted non-linear models of root growth data showed that the relationship between time and growth was highly genotype-dependent, as evidenced by the significant interaction between the spline term for date and genotype (*P* < 2.2e^−16^). Similarly, growth over time also showed significant differences across P levels (*P* = 2.141e^−07^). The three-way interaction involving time as modelled by the B-splines, genotype and treatment was found to be highly significant (*P* = 3.267e^−14^). Pairwise comparisons across genotypes showed significant differences in growth among genotypes subjected by the two different P levels, suggesting that growth patterns over time were affected by P level for three genotypes (Supplementary Data Table S1). Particularly, genotypes Crusoe, Hyking and Rubisko showed significant differences in growth pattern in the two assessed P levels (Supplementary Data Table S1).

## DISCUSSION

Root traits of 26 genotypes of bread wheat subjected to two P levels were assessed using plants grown under controlled conditions in similar trials conducted in two platforms in Italy and France. The development of high-throughput controlled-environment root screening provides a possibility to overcome the current shortage of open field options (Richards *et al*., 2010; Wasson *et al*., 2012). The results from the large-scale trial concerning biometric responses and P content in the different wheat genotypes highlighted the significant effect of genotype and P level on both shoot and root biomass, underlining the pivotal role of P in plant development. Significant genotypic variation within the same crop species has already been reported in several crops, including wheat (Deng *et al*., 2018).

### Phosphorus acquisition and use efficiency suggest distinct adaptive strategies in the tested wheat genotypes

In our work we used classical variables to assess the efficiency of plants to acquire (PAE) and use (PUE) P from the substrate, together with the efficiency of the compartment, i.e. roots, responsible for P acquisition (PRAE). The PAE is deemed to be mainly influenced by three factors: root system architecture, root exudates and interaction with soil microbes. First, root system architecture involves traits like higher root density and increased lateral root branching, which allow plants to absorb more P from the topsoil (Hammond *et al*., 2009; George *et al*., 2011; Lynch, 2011, 2019; White *et al*., 2013; Jia *et al*., 2018; Sun *et al*., 2018; Wang *et al*., 2023). Second, root exudation of protons, acids such as citrate, organic anions and enzymes is known to aid plants in releasing P from the soil (Pang *et al*., 2018; Robles-Aguilar *et al*., 2019; Wen *et al*., 2019). Third, interactions with microorganisms, like arbuscular mycorrhizal fungi that extend the soil area for P uptake (Zhang *et al*., 2018; Fang *et al*., 2024) and phosphate-solubilizing bacteria that enhance P availability in the soil (Zhang *et al*., 2016), are pivotal in changing the PAE of crops.

In the tested controlled conditions at ALSIA, regarding PAE, in addition to CIMMYT genotypes, Hyking, Ionesco, Alessio and Soissons were the most efficient under low P conditions. The genotype Hyking showed efficiency in absorbing phosphorus (PAE) even in conditions with a high P level, suggesting a good ability to assimilate P both from environments lacking in this nutrient and in optimal conditions. The PUE and PRAE values both increased with P availability for all genotypes. The contrasting correlations between PAE and PUE under P+ and P− conditions may suggest differences in how genotypes manage P availability. Under P− conditions, a positive correlation between PAE and PUE was observed in both platforms, indicating that when P is limited, plants may be under selective pressure to both acquire and utilize P efficiently. This coordination between acquisition and use under low P conditions could reflect an adaptive strategy where plants optimize both processes to cope with P scarcity. In other words, under P scarcity both processes may work in concert to optimize P management. Conversely, under P+ conditions, while a positive correlation was found in 4PMI, a negative correlation between PAE and PUE in ALSIA suggests that when the available P amount is optimal and/or abundant, genotypes that focus on acquiring large amounts of P are less efficient at using it for growth or biomass production, potentially leading to inefficiencies in internal P utilization. Considering the different trial period of the plants on the two platforms, it could be hypothesized that as plants transition from early vegetative to later stages, their physiological priorities change. Early growth stages, observed at 28 d (4PMI) might show a positive correlation as PAE directly supports PUE necessary for growth. Over a longer period, i.e. 96 d (ALSIA), as the plant matures and experiences changes in environmental conditions, this correlation between PAE and PUE might be inverted. Thus, under conditions of P abundance, after long periods acquisition may come at the expense of use efficiency. Interestingly, in rice, it has been suggested that in low P environments improvements in PUE are ideal because they do not contribute to further P mining and depletion, while targeting PAE is regarded as offering greater productivity gains, as genotypes with high PAE can maximize returns from small P fertilizer applications (Vandamme *et al*., 2016).

### Agronomic P use efficiency allowed identification of the most efficient and responsive genotypes to different P levels

Together with the four above-mentioned indicators, i.e. PAE, PUE, PRAE and PRE, additional classification into groups based on APUE values further refined the genotype selection process by combining PUE with responsiveness to P availability. This classification system can help breeders in selecting wheat genotypes that not only grow well under P-deficient conditions but also respond well to P fertilization. Based on APUE values from the ALSIA platform, and dry weight of shoot biomass, the genotypes were categorized into four groups, ER, ENR, NER and NENR, as in Neto *et al*. (2016). Ideally, efficient genotypes that generate significant shoot dry matter under low P conditions and show a strong response to added P, falling into the ER group, should be preferred for use in the field.

At ALSIA the genotypes CIMMYT15 and Alessio and at 4PMI additional genotypes (Soissons, RGT_Libravo, Gedser, Hyking) were in the ER sector, while ALESSIO was in the ENR sector. Being ranked in the ER and ENR sectors constitutes a useful selection criterion for breeding programmes (Neto *et al*., 2016). In addition, at ALSIA, among the genotypes classified as ENR due to their enhanced shoot biomass under P− conditions but low APUE values, CIMMYT4, CIMMYT10, CIMMYT11 and CIMMYT12 were included. At 4PMI, genotypes CIMMYT4, CIMMYT10 and CIMMYT12 were also found in this sector. The fact that most CIMMYT-developed genotypes fell into this category highlighted the success of their breeding strategies in enhancing their PUE. Specifically, CIMMYT15 and CIMMYT10 also showed consistent root biomass across different P levels, suggesting high tolerance to P-deficient conditions.

### Below-ground compartment is a key player in P deficiency tolerance

The root phenotyping data at ALSIA highlighted the importance of below-ground traits in the overall performance of the wheat genotypes under variable P conditions. Root growth data indicated a genotype-specific response to P levels. For instance, genotypes like Advisor and Crusoe showed pronounced growth rates under P− conditions. This could be a result of increased root elongation or branching to explore a greater soil volume to enhance P mining. These results appear consistent with those recently reported in the study by Li *et al*. (2024), which investigated the root traits of 100 winter wheat genotypes under different stress conditions, such as low P and drought, using a semi-hydroponic system. The study found that low P stresses affect root morphology, with an increase in root length and surface area, which enhances the ability of tested wheat genotypes to absorb the limited available P from the substrate (Li *et al*., 2024). In our study, the acceleration in growth during the later stages for some genotypes under P− conditions might indicate a delayed response to P deficiency, where plants allocate more resources to root development over time as a compensatory mechanism (Hermans *et al*., 2006; Richardson *et al*., 2009). Conversely, other genotypes, such as Rubisko, displayed increased root growth also in P+ conditions, suggesting less priority in resource allocation under P stress, possibly due to a trade-off with other physiological processes or an inherent inefficiency in P utilization. Among the CIMMYT lines, root growth under low P was generally reduced compared with high P conditions, but some genotypes, such as CIMMYT15 and CIMMYT12, maintained progressive root development under P deficiency. This might suggest a degree of root resilience and highlights these lines as potential donors of adaptive traits for P efficiency.

It could be hypothesized that non-efficient genotypes, which showed a reduced shoot biomass under P deficiency and an increase in root area under the same conditions, might compensate by allocating more energy towards root growth compared with shoots to enhance P uptake from the soil. On the other hand, this reallocation may not be merely adaptive, but rather driven by metabolic constraints: under low P, energy-intensive processes such as cell division and leaf expansion are limited, potentially resulting in excess soluble sugars in leaves, which can then be redirected towards less demanding compartments, i.e. roots. Phosphorus deficiency is indeed known to affect photosynthesis, sugar metabolism and carbohydrate allocation between source and sink tissues (Hermans *et al*., 2006; Xiao *et al*., 2024). The severity of P deficiency plays a crucial role in determining what adaptive root responses are induced. It has been reported that a critical decrease in soil P can initially trigger more robust root growth compared with conditions of P sufficiency (Liu, 2021; Lu *et al*., 2024). In this scenario, it has been suggested that there are two strategies adopted by plant genotypes to cope with low P conditions: a strategy based on resource acquisition, with plants showing longer total root length and lower root total density to enhance absorptive surface for P foraging from a large soil volume, and a strategy based on resource conservation, with plants having stronger root exudation per unit of the root system to explore a smaller soil volume for P more thoroughly (Wang *et al*., 2023). In line with the strategies proposed by Wang *et al*. (2023), our root phenotyping data suggest that genotypes such as Advisor, Crusoe, Donator and some CIMMYT genotypes may adopt a foraging-based strategy under P deficiency, characterized by extended root growth and exploration. In contrast, genotypes such as Rubisko and Soissons, which showed limited root expansion under P−, might have a conservation-based strategy, likely involving greater root exudation instead of root growth.

It is also worth noting that the response of root growth to P deficiency might vary depending on factors such as the P form and supply level, growth medium, soil type, sampling time, plant species and genotype (Liu, 2021). Evidence from wheat indicates that in the early growth stages shoot growth is more immediately affected by P deficiency than root growth, which continues despite low P levels, serving as an adaptive response to the P scarcity (Shen *et al*., 2018). In wheat, da Silva *et al*. (2016) observed a negative correlation between root volume and PAE (defined in their paper as PUpE), suggesting that the ability of a root system to acquire P effectively is more critical than its size. A previous root analysis of two different wheat genotypes showed that the expansion of the root system and increased development of root hairs under low P conditions enable genotypes with smaller root systems (in terms of root dry matter) to support higher yield production, demonstrating their efficiency in P utilization (Nguyen and Stangoulis, 2019). In other words, it has been hypothesized that in wheat a more efficient root system, rather than a larger one, is advantageous for better P acquisition and utilization, making it a crucial trait for plant breeders to focus on (Nguyen and Stangoulis, 2019). Notably, Yuan *et al*. (2016) observed that P deficiency simultaneously increased root length density and decreased root biomass, with the degree of these changes varying according to different soil types. On the other hand, it is worth noting that inhibition of primary root elongation and decrease in root biomass may not be solely attributable to P starvation. As previously reported in *Arabidopsis* (Ward *et al*., 2008), the presence of soluble iron can induce localized iron toxicity at the root tip under P deficiency conditions. Considering that the Hoagland solutions used in our experiments contained a soluble and available iron source (KFeEDTA), it could be argued that Fe-induced toxicity contributed to the observed root growth phenotype under P− conditions. Further experiments using reduced Fe concentrations will help to clarify the specific cross-talk between P deficiency and Fe toxicity, as well as to understand if manipulation of Fe availability in *Triticum* may represent a strategy to improve tolerance to low P conditions. Moreover, some additional analyses will make it possible to define which root system architecture trait or function is modified according to P availability and how it plays a role in plant performance. This will be made possible due to the number of root system traits that are analysed on the 4PMI platform and will be analysed in a further study.

### Similarities and differences between the two phenotyping platforms

As seen in the data, whatever growth conditions among platforms have been designed to be similar there are still some differences in the protocol that were adopted for each of the platforms. These differences are likely to be responsible for the differences in the estimated indicators as well as the different ranking between genotypes. The major difference was in the length of the experimental period, i.e. 96 d at ALSIA versus 28 d at 4PMI. The Rhizotube design of 4PMI does not allow experiments to be conducted for an extended period of time, as after some weeks the roots are entangled at the bottom of the Rhizotube and image analysis is no longer possible (Jeudy *et al*., 2016). The extended growth period at ALSIA probably allowed more extensive root development, increased P acquisition, and different PUE in response to longer-term nutrient dynamics. According to Lynch (2011), root system architecture and PUE evolve over time, with longer periods enabling roots to explore more soil and interact more extensively with soil microorganisms that facilitate P acquisition.

Additionally, the substrate that was used differed among platforms. At ALSIA, Rhizotubes were filled with P-free soil, while at 4PMI, due to the need for homogeneity throughout the substrate profile and difficulties arising when using a natural soil, the Rhizotubes were filled with an inert substrate (Jeudy *et al*., 2016). The P-free soil differs from inert substrates in terms of texture, porosity, water retention and nutrient-binding capacity. Soils have a specific P absorption capacity, as P can be bound to soil particles and be unavailable for plant uptake (Hinsinger, 2001). These binding interactions would be absent or minimal in the inert substrate used at 4PMI, where P was more likely to be freely available, potentially leading to differences in the estimation of PAE and PUE.

A third main difference between ALSIA and 4PMI was about the P supply. At ALSIA the P supply was ensured at the beginning of the experiment by adding the P only once in the soil, while at 4PMI P was supplied to the desired conditions throughout the experiment. Consequently, the substrate used in our experiment certainly modulated P availability and might be responsible for the observed differences. An alternative approach for 4PMI would be to use a slow-release method, such as P pre-adsorbed onto activated alumina and mixed into pure quartz sand, as suggested by Coltman *et al*. (1982), potentially minimizing the differences observed between platforms.

However, there were also similarities between the platforms in terms of a synthetic indicator of agronomic relevance, which allowed comparison across platforms. While the ranking of genotypes differed between ALSIA and 4PMI, ∼30 % of the genotypes were ranked similarly across platforms. Both Pearson correlation and CCC demonstrated coherence between the P indicator values from ALSIA and 4PMI. Additionally, the Bland–Altman plot confirmed these findings, with only the Pibrac genotype falling outside the upper and lower limits of agreement. This suggests a high level of consistency across platforms as well as supporting the use of the synthetic P indicator for comparison and ranking of genotypes. Thus, the proposed P indicator, embedding information from five different indicators of efficiency in P uptake and use by plants, can be of agronomic relevance not only to researchers working in phenotyping facilities for assessing P deficiency tolerance in wheat genotypes, but also to breeders who need a simplified and effective tool for selecting and ranking genotypes based on their P efficiency in different growing conditions. Selection of P-deficiency-tolerant genotypes is pivotal, since domestication and commercial breeding in high-nutrient soils have resulted in plants that have lost specific root traits important for tolerating low-P soils, making them suitable only for the modern, intensive agricultural systems with high input of mineral fertilizers (Wang *et al*., 2023).

### Conclusions

Our study assessed P acquisition and use efficiencies across bread wheat genotypes using two distinct phenotyping platforms, 4PMI and ALSIA, under controlled conditions and different P levels. Under a high P level and over a long period, high acquisition efficiency may come at the expense of use efficiency. Conversely, under low P, genotypes demonstrated a positive correlation between PAE and PUE, suggesting optimized acquisition and use through root adaptations in P-limited environments. The proposed synthetic P indicator, integrating five efficiency metrics, demonstrated consistent performance across platforms, confirmed by correlation and concordance correlation values. This indicator may provide a practical tool for breeders, allowing ranking of genotypes for P efficiency, especially under P-deficient conditions. Overall, these findings highlight root-driven P acquisition as a key adaptive strategy and support using multitrait selection to improve P acquisition and use efficiencies in wheat, advancing sustainable agricultural practices.

## Supplementary Material

mcaf091_Supplementary_Data

## SUPPLEMENTARY DATA

Supplementary data are available at *Annals of Botany* online and consist of the following. Table S1: pairwise comparisons of root growth areas of wheat genotypes grown under P− and P+ conditions at the ALSIA platform. Figure S1: P content in plant tissues under P+ and P− conditions across the wheat genotypes grown at 4PMI (top panel) and ALSIA (bottom panel). Figure S2: Bland–Altman plot of mean P indicator values comparing ALSIA and 4PMI platforms. Figure S3: correlation between P efficiency indices measured across bread wheat genotypes at ALSIA and 4PMI. Video S1: segm ented shoot and root images for Bagou under P+ and P− conditions at 4PMI.
