# Supplementary material for: A novel P indicator to evaluate bread wheat (Triticum aestivum) genotypes to identify tolerance to phosphorus deficiency based on two distinct root phenotyping platforms
Source: Ann Bot. 2025 Jun 10;136(5-6):1203–18. doi: 10.1093/aob/mcaf091 (PMC12682855; doi:10.1093/aob/mcaf091)
Supplement: mcaf091_Supplementary_Data [file mcaf091_supplementary_data.zip › aob-25161-s03.docx]

**Table S1.** Pairwise comparisons of root growth areas of wheat genotypes grown under P- and P+ conditions in ALSIA platform. T-ratios and p-values (BH adjusted) are reported to show the statistical significance of the “P level” effect within each genotype.

| **Genotype** | **Comparison** | **t-ratio** | **p-value** |
| --- | --- | --- | --- |
| ADVISOR | P- vs P+ | 0.863 | 0.3887 |
| ALESSIO | P- vs P+ | 0.026 | 0.9792 |
| ANNECY | P- vs P+ | -0.783 | 0.4338 |
| ARMADA | P- vs P+ | -0.959 | 0.3380 |
| BAGOU | P- vs P+ | 0.721 | 0.4710 |
| CALIXO | P- vs P+ | 0.048 | 0.9615 |
| CIMMYT10 | P- vs P+ | 0.921 | 0.3576 |
| CIMMYT11 | P- vs P+ | 0.259 | 0.7954 |
| CIMMYT12 | P- vs P+ | -0.413 | 0.6797 |
| CIMMYT15 | P- vs P+ | -0.325 | 0.7452 |
| CIMMYT4 | P- vs P+ | -1.591 | 0.1123 |
| COMPLICE | P- vs P+ | 0.492 | 0.6231 |
| CRUSOE | P- vs P+ | 1.904 | 0.0498* |
| DONATOR | P- vs P+ | 1.032 | 0.3025 |
| FOXYL | P- vs P+ | -1.457 | 0.1457 |
| GEDSER | P- vs P+ | -1.354 | 0.1764 |
| HEREWARD | P- vs P+ | -1.569 | 0.1172 |
| HYKING | P- vs P+ | -3.123 | 0.0019** |
| IONESCO | P- vs P+ | 1.141 | 0.1882 |
| JOHNSON | P- vs P+ | -0.634 | 0.5263 |
| PIBRAC | P- vs P+ | -1.221 | 0.2225 |
| RGT LEXIO | P- vs P+ | -0.510 | 0.6102 |
| RGT LIBRAVO | P- vs P+ | -1.623 | 0.1051 |
| ROBIGUS | P- vs P+ | 0.650 | 0.5160 |
| RUBISKO | P- vs P+ | -1.902 | 0.0504* |
| SOISSONS | P- vs P+ | -0.397 | 0.6918 |
