# Supplementary material for: A novel P indicator to evaluate bread wheat (Triticum aestivum) genotypes to identify tolerance to phosphorus deficiency based on two distinct root phenotyping platforms
Source: Ann Bot. 2025 Jun 10;136(5-6):1203–18. doi: 10.1093/aob/mcaf091 (PMC12682855; doi:10.1093/aob/mcaf091)
Supplement: mcaf091_Supplementary_Data [file mcaf091_supplementary_data.zip › Supplementary_Legends.docx]

Supplementary data are available online at https://academic.oup.com/aob and consist of the following:

**Table S1.** Pairwise comparisons of root growth areas of wheat genotypes grown under P- and P+ conditions in ALSIA platform.

**Figure S1.** Phosphorus content in plant tissues under P+ and P- conditions across the wheat genotypes grown at 4PMI (top panel) and ALSIA (bottom panel).

**Figure S2.** Bland-Altman plot of mean P indicator values comparing ALSIA and 4PMI platforms.

**Figure S3.** Correlation between P efficiency indices measured across bread wheat genotypes at ALSIA and 4PMI.

**Video S1.** Segmented shoot and root images for Bagou under P+ and P- from 4PMI platform.
