# Supplementary figures and images for: A novel P indicator to evaluate bread wheat (Triticum aestivum) genotypes to identify tolerance to phosphorus deficiency based on two distinct root phenotyping platforms
Source: Ann Bot. 2025 Jun 10;136(5-6):1203–18. doi: 10.1093/aob/mcaf091 (PMC12682855; doi:10.1093/aob/mcaf091)

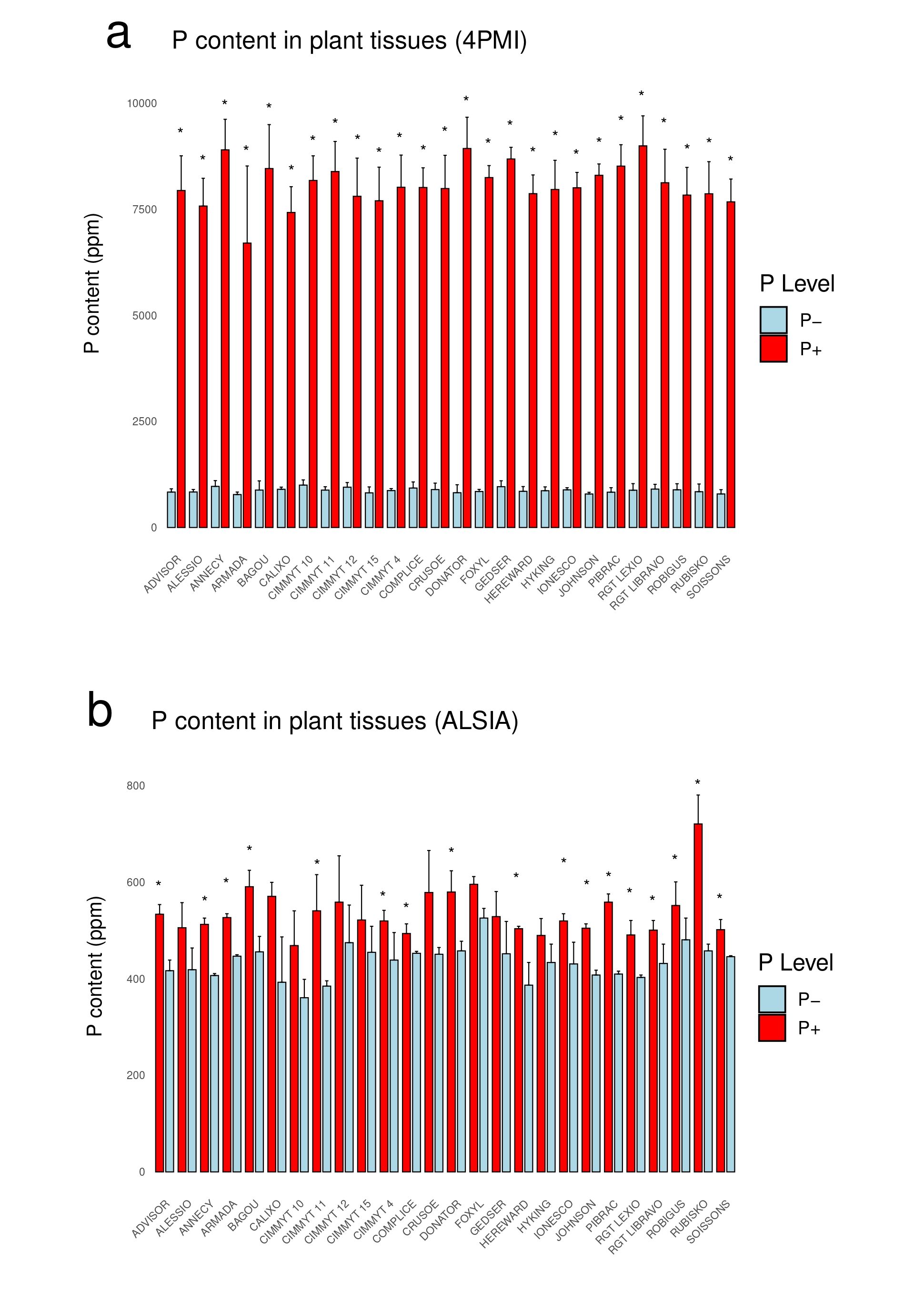

Supplement: mcaf091_Supplementary_Data [file mcaf091_supplementary_data.zip › aob-25161-s04.jpg]

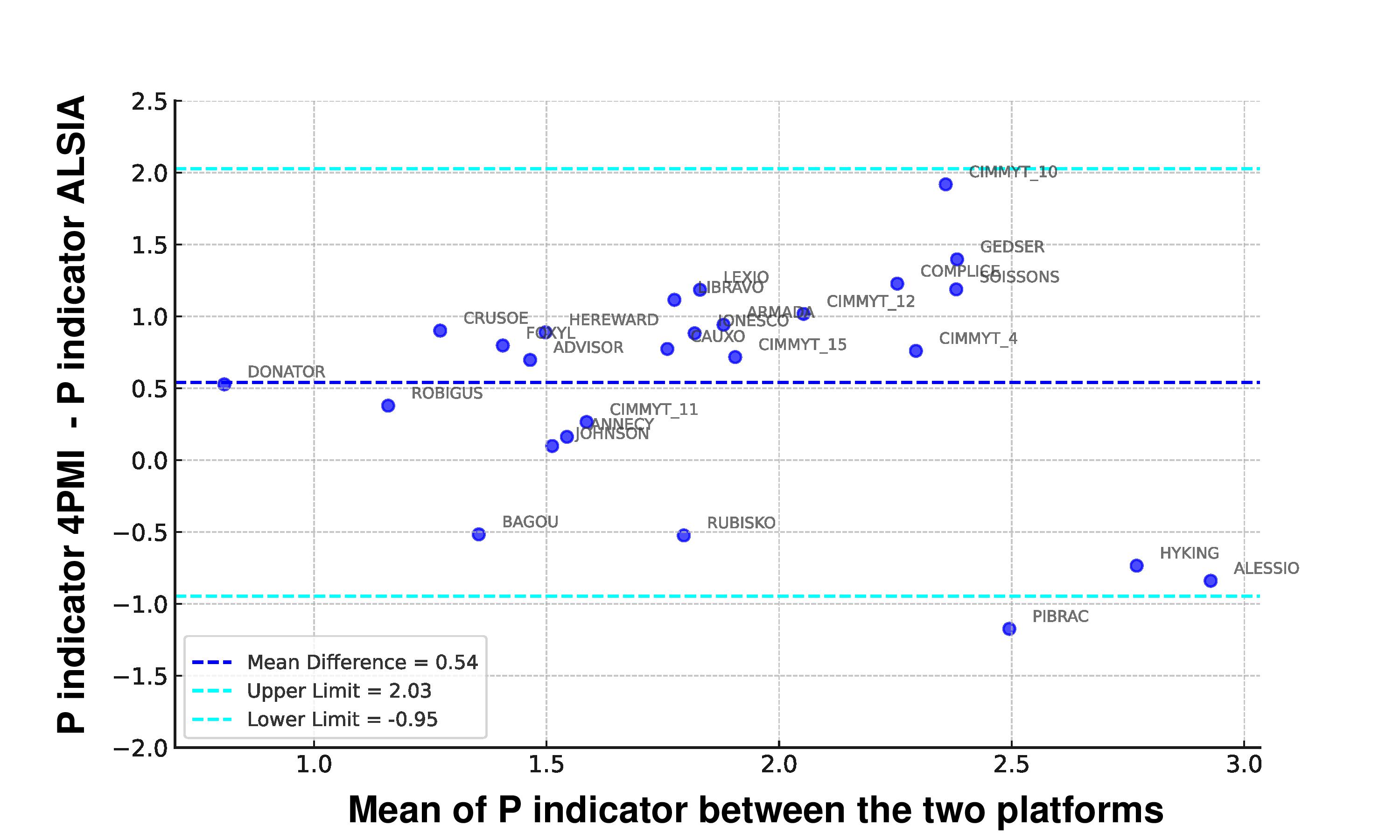

Supplement: mcaf091_Supplementary_Data [file mcaf091_supplementary_data.zip › aob-25161-s05.jpg]

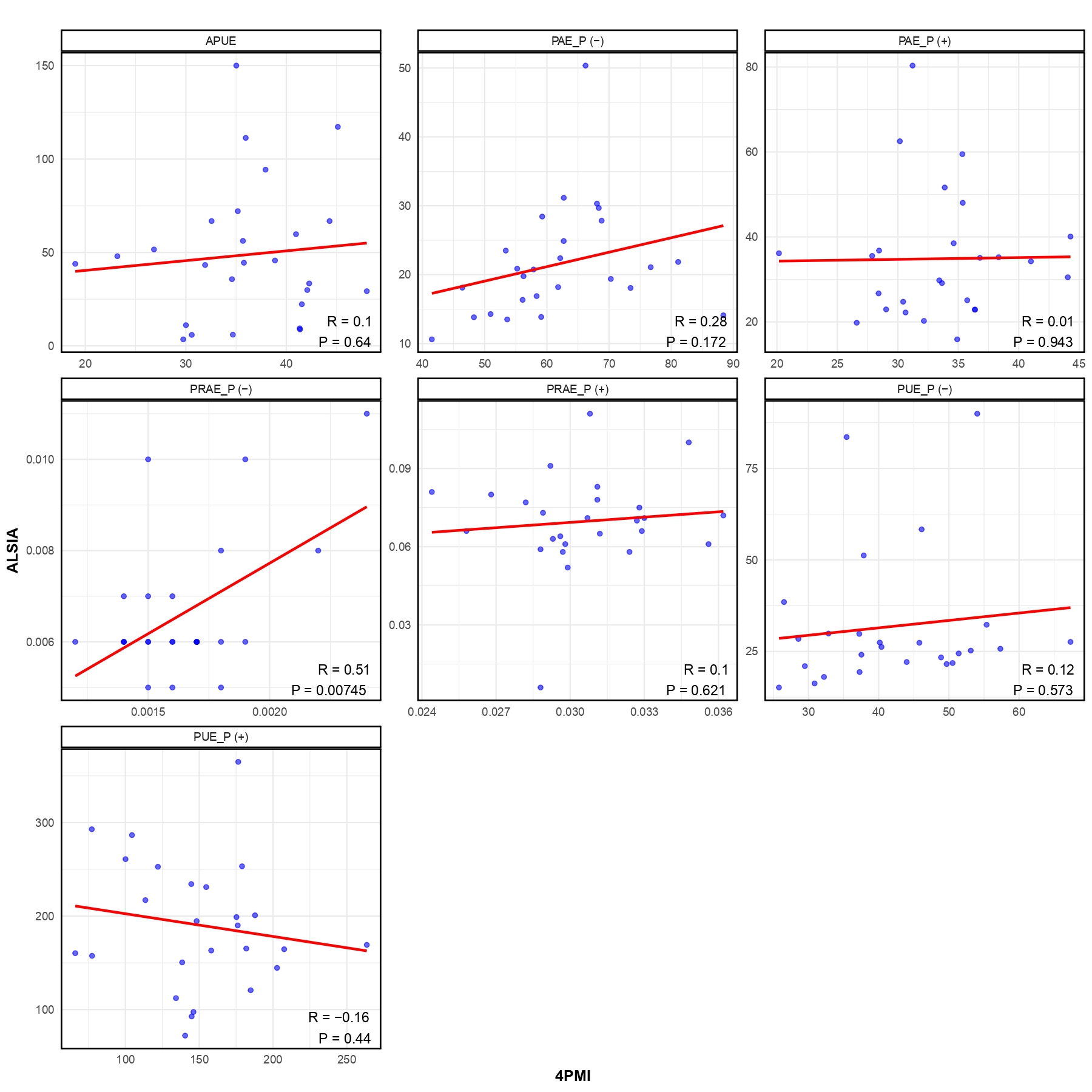

Supplement: mcaf091_Supplementary_Data [file mcaf091_supplementary_data.zip › aob-25161-s06.jpg]
